# Insights Into the Role of Heat Shock Protein 27 in the Development of Neurodegeneration

**DOI:** 10.3389/fnmol.2022.868089

**Published:** 2022-03-30

**Authors:** Bianka A. Holguin, Zacariah L. Hildenbrand, Ricardo A. Bernal

**Affiliations:** Department of Chemistry and Biochemistry, University of Texas at El Paso, El Paso, TX, United States

**Keywords:** small heat shock protein 27 (Hsp27), Charcot-Marie-Tooth disease (CMT), distal hereditary motor neuropathy (dHMN), α-crystallin domain (ACD), heat shock protein

## Abstract

Small heat shock protein 27 is a critically important chaperone, that plays a key role in several essential and varied physiological processes. These include thermotolerance, apoptosis, cytoskeletal dynamics, cell differentiation, protein folding, among others. Despite its relatively small size and intrinsically disordered termini, it forms large and polydisperse oligomers that are in equilibrium with dimers. This equilibrium is driven by transient interactions between the N-terminal region, the α-crystallin domain, and the C-terminal region. The continuous redistribution of binding partners results in a conformationally dynamic protein that allows it to adapt to different functions where substrate capture is required. However, the intrinsic disorder of the amino and carboxy terminal regions and subsequent conformational variability has made structural investigations challenging. Because heat shock protein 27 is critical for so many key cellular functions, it is not surprising that it also has been linked to human disease. Charcot-Marie-Tooth and distal hereditary motor neuropathy are examples of neurodegenerative disorders that arise from single point mutations in heat shock protein 27. The development of possible treatments, however, depends on our understanding of its normal function at the molecular level so we might be able to understand how mutations manifest as disease. This review will summarize recent reports describing investigations into the structurally elusive regions of Hsp27. Recent insights begin to provide the required context to explain the relationship between a mutation and the resulting loss or gain of function that leads to Charcot-Marie Tooth disease and distal hereditary motor neuropathy.

## Introduction

The proteome is a vast collection of proteins each of which contributes to the maintenance of cellular homeostasis (Kim et al., 2013; Bakthisaran et al., 2015; Webster et al., 2019). Diverse environmental and physiological stressors can lead to various deleterious effects including structural damage of proteins (Sun and MacRae, 2005; Kim et al., 2013; Webster et al., 2019). With so many threats overwhelming the integrity of the proteome it is imperative that the cell ensures a robust defense mechanism to keep its proteins structurally viable and functional (Eyles and Gierasch, 2010; Kim et al., 2013). Cellular stressors such as heat shock can often result in partial protein misfolding that can then lead to various disorders including neurodegeneration (Eyles and Gierasch, 2010; Webster et al., 2019). The integrity of protein structure and function is maintained by a class of proteins known as molecular chaperones (Sun and MacRae, 2005; Bhatt et al., 2018; Rodriguez et al., 2020). These molecular chaperones sustain protein homeostasis through assistance with protein folding or the stabilization of partially misfolded proteins (Sun and MacRae, 2005; Kim et al., 2013). A subclass of molecular chaperones, known as heat shock proteins, can assist in the folding of nascent polypeptides or aid in the stabilization and the refolding of non-native proteins (van Montfort et al., 2001; Fu, 2014; Enriquez et al., 2017; Bhatt et al., 2018). They are also involved in other pathways such as degradation of irreversibly damaged proteins and unfolding of other proteins for translocation across a membrane (Haslbeck et al., 1999; Shashidharamurthy et al., 2005; Richter et al., 2010).

Heat shock proteins are broadly conserved amongst six major classes. These include Hsp100, Hsp90, Hsp70, Hsp60, Hsp40, and the small heat shock proteins (Sun and MacRae, 2005; Richter et al., 2010; Fu, 2014; Bakthisaran et al., 2015; Enriquez et al., 2017; Rodriguez et al., 2020). Small heat shock proteins (sHsps) are molecular chaperones that do not require the hydrolysis of ATP (Bova et al., 2002; Sobott et al., 2002). Their primary role is to maintain a partially denatured protein in a folding-competent state to protect it from irreversible and detrimental aggregation (Borrelli et al., 2002; Bakthisaran et al., 2015). Small heat shock protein 27 is a ubiquitously expressed chaperone involved in many physiological and metabolic pathways (Parcellier et al., 2005). In addition to protein folding, it has been implicated in the regulation of redox states, stabilization of the cytoskeleton, anti-apoptotic activity, and in important interactions with transcription factors and mRNA processing proteins (D’Ydewalle et al., 2011; Chalova et al., 2014; Fu, 2014). Hsp27 (HspB1) belongs to the subgroup of small heat shock proteins that encompass 10 human sHsps. These small heat shock proteins are encoded by the genes HspB1–10 and have been categorized into two main classes (Bakthisaran et al., 2015; Muranova et al., 2015; Webster et al., 2019). Class I sHsp include HspB1 (Hsp27), HspB5 (α-B-crystallin), HspB6 (Hsp20), and HspB8 (Hsp22; Kappe et al., 2003; Bakthisaran et al., 2015). These sHsps are widely distributed with expression in nearly all cells and tissues where they perform various roles (Kappe et al., 2003; Mainz et al., 2015; Mymrikov et al., 2017). The second class of small heat shock proteins includes HspB2, HspB3, HspB4 (αA-crystallin), HspB7 (cvHsp), HspB9, and HspB10 (ODF1) and are restricted to certain types of tissues such as the eyes, heart, and testes (Kappe et al., 2003; Bakthisaran et al., 2015).

**Figure 1:**
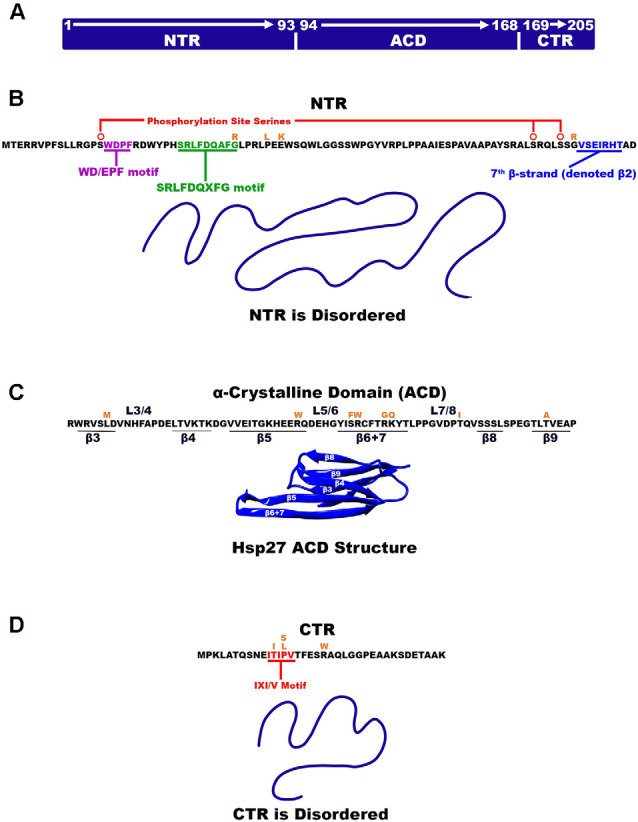
Hsp27 Structural organization. **(A)** Hsp27 is made up of three regions where each region is identified as NTR, ACD, and CTR. The residue numbers that make up each region are also denoted. **(B)** The amino acid sequence of the disordered NTR is listed and the two motifs WD/EPF and SRLFDQxFG are highlighted with red and green, respectively. β-strand β-2 is highlighted in cyan. **(C)** The ACD amino acid sequence is listed as well as the location of β-strands and their connecting loops. The ACD is the only part of Hsp27 that is ordered (PDB 2N3J). **(D)** Hsp27 disordered CTR highlighting the IXI/V motif in red. Amino acid mutations are illustrated in orange above the amino acid being mutated.

**Figure 2:**
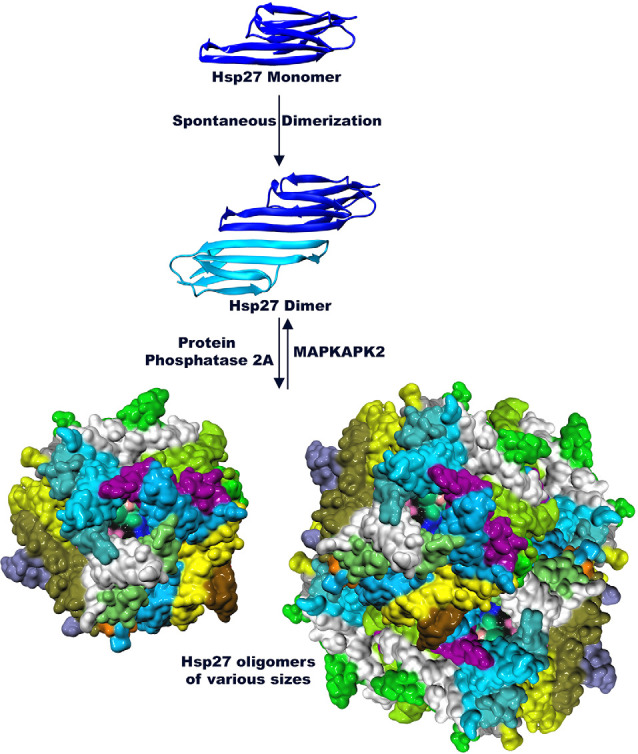
Hsp27 is organized into higher order structures (PDB 6DV5 and 2N3J). The monomer of Hsp27 assembles into dimers *via* the ACD. Dimers in turn interact with each other to form large highly dynamic multimers. Phosphorylation by MAPKAPK2 dissociates oligomers of Hsp27 and favors dimer formation while Protein Phosphatase 2A shifts the equilibrium towards multimer formation.

Small heat shock proteins are characterized by a conserved sequence of approximately 80–100 residues known as the α-crystallin domain (ACD; Fu, 2014; Bakthisaran et al., 2015; Muranova et al., 2015; Clouser et al., 2019). The ACD is flanked by a variable hydrophobic amino terminal region (NTR) and a short flexible c-terminal region (CTR; Garrido et al., 2012; Hochberg et al., 2014; Rajagopal et al., 2015; Figure 1). Small heat shock proteins form dimers *via* β-sheets formed by the ACD. These dimers serve as building blocks of class 1 sHsp that organize into large oligomeric complexes that are stabilized by their N-terminal and C-terminal regions (Lelj-Garolla and Mauk, 2012; McDonald et al., 2012; Aquilina et al., 2013; Rajagopal et al., 2015). The dimers are thought to be the smallest functional form of small heat shock proteins (Lambert et al., 1999; Hayes et al., 2009). These complexes engage in rapid dimer exchange creating a dynamic equilibrium between large multimers of various sizes and dimers (Bova et al., 2000, 2002; Sobott et al., 2002; Rajagopal et al., 2015; Figure 2). The stress pathway activates p38 mitogen-activated protein kinase, which in turn phosphorylates mitogen-activated protein kinase activated protein kinase (MAPKAPK2; Kostenko and Moens, 2009; Bakthisaran et al., 2015; Lang et al., 2021). Phosphorylation by MAPKAPK2 shifts the equilibrium to small oligomers and dimers whereas protein phosphatase 2A activity reverses this process and favors the formation of large multimers (Cairns et al., 1994; Kostenko and Moens, 2009; Katsogiannou et al., 2014; Bakthisaran et al., 2015; Figure 2). This system might be a way to store large amounts of Hsp27 to quickly react to stress without the need for *de novo* protein synthesis (Peschek et al., 2013).

Small heat shock proteins can be perturbed by a myriad of inherited and sporadic mutations that result in the formation of neurological disorders. For example, various missense mutations in the small heat shock protein 27 (Hsp27) cause the neuropathies Charcot-Marie-Tooth Disease Axonal Type 2F (CMT2F) and Distal Hereditary Motor Neuropathy 2B (dHMN2B; Houlden et al., 2008; Tanabe et al., 2018). These neuropathies are commonly inherited disorders that exclusively affect the peripheral nervous system (Holmgren et al., 2013). They are closely related in their key features of disease progression and only differ by the point mutation that leads to the respective disease. CMT2F is a subset of the more broadly categorized forms of CMT, specifically characterized by axonal degeneration of the peripheral nerves (Ismailov et al., 2001; Schwartz, 2019). The characteristic symptom of CMT2F includes the slow progressive loss of motor function manifested as weakness and muscle atrophy of the distal limbs (Evgrafov et al., 2004; Gentile et al., 2020). Additionally, there can be decreased tendon reflexes of the ankle and legs, as well as physical deformities of the feet (Webster et al., 2019; Gentile et al., 2020). In cases of sensory neuron involvement, afflicted individuals lose pain and temperature sensation (Szigeti and Lupski, 2009). The disease, although progressive over many years, does not affect the life span (Schwartz, 2019). Individuals with CMT2F typically experience the onset of symptoms during adulthood and may require treatment through physical therapy, mobility aids, orthopedic shoes, and/or surgery (Evgrafov et al., 2004). More severe forms of CMT2F can result in hearing loss and vocal cord impairment, in addition to acute distal limb pain (Bakthisaran et al., 2015).

While CMT2F manifests as a motor and sensory neuropathy, Distal Hereditary Motor Neuropathy Type II (dHMNIIB) exclusively affects motor neurons (Geuens et al., 2017; Schwartz, 2019). dHMNIIB clinically presents similarly to CMT2F as they are related diseases caused by some of the same mutations (Tanabe et al., 2018). Individuals experience lower limb weakness due to muscle loss and deteriorating leg mobility (Dierick et al., 2007; Chung et al., 2008; Tanabe et al., 2018). The typical onset of the disease occurs in individuals between the ages of 15 and 45 and is a progressive disease that severely compromises the quality of life (Dierick et al., 2007; Chung et al., 2008; Schwartz, 2019). Both CMT2F and dHMNIIB have autosomal dominant patterns of inheritance indicating that only one copy of a mutated gene is sufficient to cause disease (Evgrafov et al., 2004; Schwartz, 2019). These neuropathies are described as being clinically and genetically heterogenous (Ackerley et al., 2006; Dierick et al., 2007; Echaniz-Laguna, 2015; Tanabe et al., 2018). This means that the same mutation can result in different clinical presentations and different mutations can manifest as the same disease, further complicating the understanding of the pathomechanisms. There are many mutations in Hsp27 that have been implicated in hereditary neuropathies (Muranova et al., 2019; Vendredy et al., 2020). A number of these mutations result in either CMT2F, dHMN2B, or both. There have been several reports linking mutated Hsp27 to disturbances in the cytoskeletal components of axons (Almeida-Souza et al., 2011; D’Ydewalle et al., 2011; Nefedova et al., 2017). Several substrates of Hsp27, such as Tau and α-synuclein, are involved in stabilizing cytoskeletal components and vesicle trafficking (Abisambra et al., 2010; Barbier et al., 2019; Baughman et al., 2020). These are processes that are required for normal motor neuron functioning (Abisambra et al., 2010; Barbier et al., 2019). These relationships have been implicated as indirect causes for many neurodegenerative diseases including CMT2F and dHMN2B (Webster et al., 2019; Muranova et al., 2020; Vendredy et al., 2020).

Hsp27 has been suspected in the progression of other neurodegenerative disorders such as Alzheimer’s and Parkinson’s disease (Zhang et al., 2014; Hu et al., 2021). They are the top two most common neurological illnesses affecting roughly 12% of people over 60 years of age (Hu et al., 2021). Alzheimer’s disease (AD) is also a progressive neurodegenerative disorder localized to the brain, mainly affecting neurons of the cortex (Nussbaum and Ellis, 2003). The classical manifestations of Alzheimer’s are an accumulation of amyloid-β plaques and neurofibrillary tangles of hyperphosphorylated tau (Nussbaum and Ellis, 2003). Alzheimer’s is the number one cause of dementia and severely diminishes the quality of life of those affected. Parkinson’s disease (PD) results in the irreversible degeneration of dopaminergic neurons in the substantia nigra and includes the aggregation of α-synuclein contained within Lewy bodies and neuritis (Nussbaum and Ellis, 2003; Poewe et al., 2017). Parkinson’s disease, like Alzheimer’s, can also cause dementia in addition to the classical parkinsonian symptoms including tremors, rigidity, and bradykinesia (Williams and Litvan, 2013). Recently increased attention has targeted the involvement of Hsp27 in the progression of these neurological diseases from a therapeutic standpoint (Venugopal et al., 2019; Baughman et al., 2020; Vicente Miranda et al., 2020; Navarro-Zaragoza et al., 2021). The substrates of Hsp27 such as Tau and α-synuclein are major components in the pathological progression of both AD and PD. Although there is no evidence to suggest that mutations of Hsp27 are linked to AD and PD, the faulty chaperone may contribute to disease by ineffective regulation of these substrates.

### Features of the Small Heat Shock Protein 27 Regions

Three major structural regions of Hsp27 can be defined by the structural and functional distinctions between them. The N-terminal region (NTR) of Hsp27 is composed of the first 93 residues and nearly 50% of the Hsp27 primary sequence (Clouser et al., 2019; Figures 1A,B). The NTR is inherently disordered and composed of predominantly hydrophobic and aromatic residues. It is thought to be primarily responsible for driving/stabilizing higher order multimers of Hsp27 (Bakthisaran et al., 2015). A less conserved region within the NTR is the WD/EPF motif made of residues 16-WDPF-19 that have been shown to correlate with protein chaperoning activity (Thériault et al., 2004; Lelj-Garolla and Mauk, 2012). Hsp27 is phosphorylated *via* the p38 MAPK pathway by MAPKAPK2, which occurs at serine residues 15, 78, 82 of the NTR (Lambert et al., 1999; Shashidharamurthy et al., 2005; Figure 1B). The phosphorylation site at serine-15 precedes the NTR WD/EPF domain while phosphorylation sites 78 and 82 reside near the end of the NTR (Lelj-Garolla and Mauk, 2012). In its unphosphorylated state Hsp27 exists as a large polydisperse oligomeric species with a molecular weight of up to ~650 kDa (Rogalla et al., 1999). Experimental evidence has demonstrated that tunability of Hsp27 oligomeric size can be achieved through stepwise phosphorylation of the three phosphorylation sites (Hayes et al., 2009; Jovcevski et al., 2015). Phosphorylation of all three sites yields smaller oligomers and dimers whereas phosphorylation of one or two sites results in intermediately sized oligomers (Hayes et al., 2009; Jovcevski et al., 2015). At residue positions, 26–34 and near the center of the NTR is the conserved SRLFDQxFG motif (Pasta et al., 2003). Truncation studies have confirmed that this motif plays a large part in the production and stability of higher order multimers (Pasta et al., 2003). The NTR appears to be a highly flexible region that can adopt several conformations to facilitate oligomerization.

**Figure 3:**
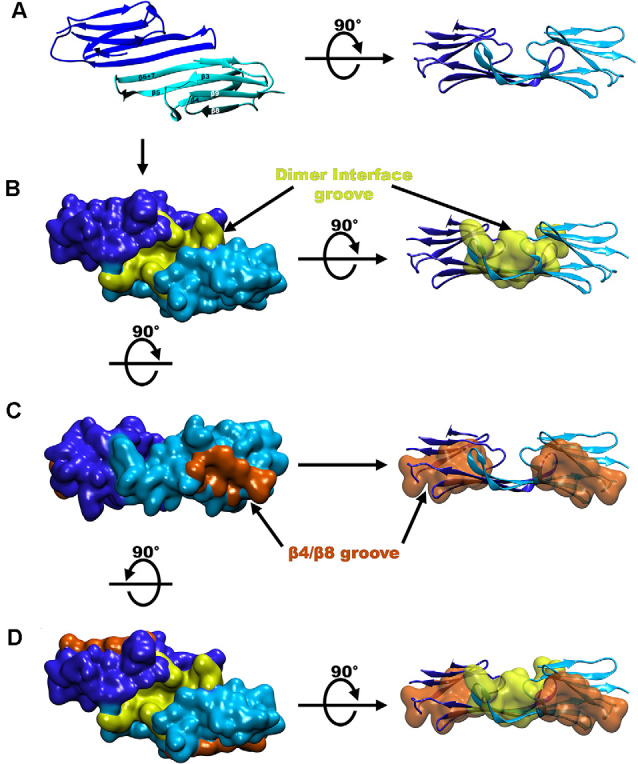
Groove locations in the crystal structure of Hsp27. **(A)** A ribbon diagram of the Hsp27 dimer (PDB 4MJH). **(B)** Hsp27 structure seen in panel **(A)** but in surface representation where the dimer interface groove is shaded yellow. **(C)** Hsp27 β4/β8 groove (colored in orange). **(D)** Representation of the three grooves on a dimeric unit colored the same as in panels **(B,C)**.

The Hsp27 α crystalline domain (ACD) is comprised of residues 94–168 that are arranged into six β-strands that form two β-sheets (Baranova et al., 2011; Alderson et al., 2019; Nappi et al., 2020; Figure 1C). This immunoglobulin-like fold of the ACD is conserved amongst various organisms in both sequence and structure (Weeks et al., 2018; Clouser et al., 2019). The β-strands denoted as 4, 5, and extended strands 6 + 7 make up one β-sheet, whereas β-strands 3, 8, and 9 form another β-sheet (Hochberg et al., 2014; Klevit, 2020). Residues on the carboxy-terminal side of the NTR (84–91) periodically form a seventh β-strand (denoted β2) that integrates into the β3/β8/β9 β-sheet (Collier et al., 2019; Klevit, 2020; Figure 1B). The driving force for Hsp27 dimerization is hydrogen bonding and electrostatic interactions between ACDs. This occurs by pairwise alignment of the extended β6 + 7 strands in an antiparallel 2 register (AP2) creating the dimer interface and an extended β-sheet (Aquilina et al., 2013; Rajagopal et al., 2015; Clouser et al., 2019; Boelens, 2020; Figure 3A). The extended β-sheet is the sum of two β6 + 7 containing β-sheets forming one longer extended symmetry related β-sheet. The extended β-sheet and two β3/β8/β9 β-sheets from each monomer create a groove that is located right above the dimer interface and between the two β3/β8/β9 β-sheets, therein described as the dimer interface groove (Klevit, 2020; Figure 3B). In total, 18 residues with their side chains pointing inward, create the dimer interface groove. Eight of these residues are from each β6 + 7 strand and 10 are from loop regions that connect the β-strands 3 and 4 (L3/4) and β-strands 5 and 6 + 7 (L5/6; Klevit, 2020).

Another structurally notable feature of the ACD is the β4/β8 groove which includes a hydrophobic pocket centered between β-strands 4 and 8 and is located on the outer edge of the ACD β-sandwich (Ehrnsperger et al., 1997; Klevit, 2020; Reid Alderson et al., 2021; Figure 3C). Therefore, within a dimer of Hsp27 there are two hydrophobic grooves located at the edges of each monomer and one shared dimer interface groove created by the linkage of two monomers (Clouser et al., 2019; Boelens, 2020; Figure 3D). These grooves provide regions for several client protein and oligomer binding opportunities and will be discussed later in this review.

The C-terminal region (CTR) and extension make up the remaining 37 residues of the Hsp27 primary sequence (Figure 1A). The CTR has an abundance of polar and charged residues and is partly responsible for oligomer formation and is thought to aid in the solubility of Hsp27 oligomers and or oligomer/substrate complexes (Pasta et al., 2004; Chalova et al., 2014; Carver et al., 2017; Boelens, 2020). The CTR contains a well-conserved region known as the IXI/V motif at residue positions 179–183 characterized by alternating isoleucine and valine residues with the central residue being a canonical proline (Lelj-Garolla and Mauk, 2012; Carver et al., 2017; Figure 1D). This motif contributes to the stabilization of oligomers by interlinking adjacent dimers. The C-terminal extension consists of the last 22 residues and does not contain any secondary structure (Alderson et al., 2017; Carver et al., 2017). The extension including the IXI/V motif is inherently disordered and highly mobile (Alderson et al., 2017). This is partly due to cis-trans isomerization about the 182 and 194 proline residues which causes changes in the adopted disordered states (Alderson et al., 2017). Interestingly, however, the residues near these pralines were shown to convert into a low residual β-strand structure which is thought to have implications in regulating oligomerization stability (Alderson et al., 2017).

### Hsp27 NTR ACD and CTR Interact to Form Higher Order Complexes

**Figure 4:**
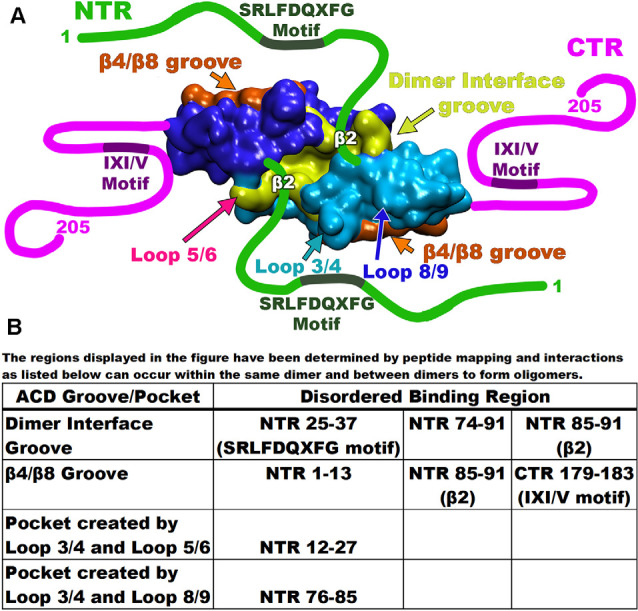
Map of Hsp27 interacting regions. **(A)** Each of the monomers is depicted as either blue or cyan with the dimer interface groove in yellow and the β4/β8 groove for each monomer in orange. Disordered regions are drawn as either green or magenta lines with the binding motifs as labeled. Important interaction loops are indicated by arrows. **(B)** The table at the bottom lists each of the disordered binding regions and the groove/pocket that bind as described by peptide mapping.

Recent studies have revealed that the involvement and dynamics of the NTR in oligomer formation is far more complicated than originally thought (Clouser et al., 2019; Collier et al., 2019; Nappi et al., 2020). Various techniques including NMR, HX-MS, and modeling were employed to characterize the NTR of Hsp27 (Clouser et al., 2019). These revealed that various hydrophobic peptide regions of the NTR make multiple contacts with two grooves of the ACD (Clouser et al., 2019). Using six truncated variations of the NTR, a map of the binding combinations of NTR and ACD displayed four separate interactions (Clouser et al., 2019). First, the distal region of the NTR that encompasses the first 13 residues interacts with the β4/β8 groove of the ACD (Figure 4). This interaction is thought to be the result of alternating hydrophobic residues on the distal region of the NTR (6-VPFSLL-11) considered to be a motif that can bind the β4/β8 groove (Clouser et al., 2019). The next set of residues of the NTR, that contains serine-15 and the WD/EPF motif (12–27), was shown to bind a negatively charged area made of loops L3/4 and L5/6 (Figure 4; Clouser et al., 2019). Interestingly, serine residues 78 and 82, although distant in sequence, were also found near this NTR binding region (Clouser et al., 2019). It is suggested that this phosphorylated serine congregation facilitates the regulation of phosphorylation (Clouser et al., 2019). Additional negative charges, introduced by phosphorylation, disturb the region, and promote dissociation of the NTR (12–27) from the negatively charged loops (Hayes et al., 2009; Clouser et al., 2019).

The remaining segments of the NTR (residues 25–37 and 74–91) are found to bind the groove created by the dimer interface (Figure 4). For example, the NTR (25–37) contains the conserved SRLFDQxFG motif (Pasta et al., 2003; Clouser et al., 2019). This motif binds at the dimer interface groove much like the NTR (74–91) that contains the phosphorylation site serine residues (Klevit, 2020). This suggests that at any given time, several regions of the NTR may be bound to different grooves within the ACD. Another study proposed an additional NTR-ACD binding combination (Collier et al., 2019). Using a previous structure and co-crystallization strategy, an ACD construct was built that included NTR phosphorylation site serines 78 and 82 (Collier et al., 2019). The β2 strand of Hsp27 (residues 84–93) transiently binds the adjacent β3 strand in an antiparallel orientation (Clouser et al., 2019; Collier et al., 2019; Klevit, 2020). The presence of the β2 strand, therefore, alters the space allotted within the dimer interface groove (Collier et al., 2019; Klevit, 2020). Various peptide mimics of the NTR crystallized with an ACD construct (84–171) confirm a hydrogen bonding network between the β2 and β3 strands (Collier et al., 2019). Additionally, a third Hsp27 binding combination has revealed that the β2 strand becomes extended and transiently binds the β4/β8 groove of an adjacent dimer (Figure 4; Collier et al., 2019). As mentioned previously, the NTR (12–27) containing serine 15, binds a space between negatively charged loops L3/4 and L5/6. This was found to perturb the phosphorylation site residues S78 and S82 indicating that the three phosphorylation sites are close (Clouser et al., 2019). This is confirmed by the crystal structure (6GJH) revealing that a peptide segment containing the phosphorylation site serines 78 and 82 binds a pocket created by loops L3/4 and L8/9 within the ACD (Figure 4; Collier et al., 2019). Therefore, the binding pockets for the NTR regions share the center L3/4 loop indicating that the phosphorylation site serines 15, 78, and 82 are close in space. Recently, an x-ray structure defined a spherical Hsp27 oligomer complex composed of 12 dimers (Nappi et al., 2020). The structure revealed that serine residues 78 and 82 from each monomer share the same area. Collectively, these studies reveal that between adjacent dimers, six phosphorylation sites are arranged near each other. This supports the claim that charge repulsion is the driving force of oligomer dissociation that results in a molecule with increased flexibility (Collier et al., 2019).

**Figure 5:**
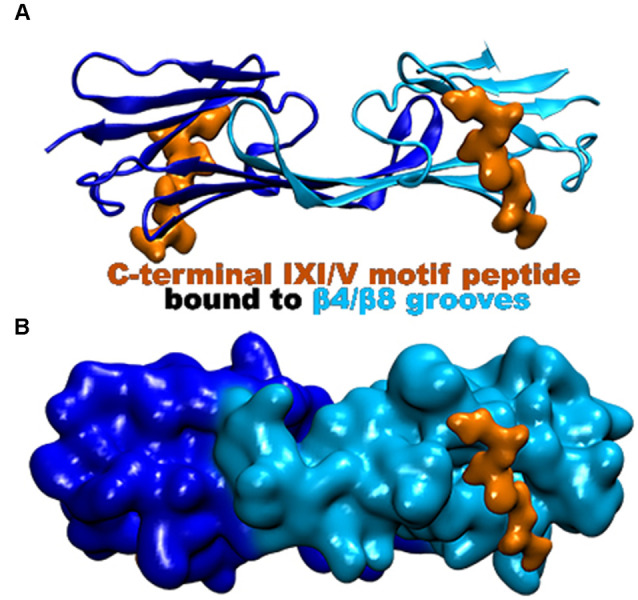
Hsp27 crystal structure (PDB 4MJH) co-crystallized with peptide mimic of the IXI/V motif. The peptide (colored in orange) was found bound to the β4/β8 groove as indicated. **(A)** Ribbon representation of the dimer illustrating the location of the two bound peptides. **(B)** Surface representation of the dimer that better illustrates the β4/β8 groove with the bound peptide.

The CTR of the small heat shock proteins forms transient interactions with regions on the ACD (Pasta et al., 2004). As seen with many sHsp the IXI/V motif within the CTR fits into the hydrophobic β4/β8 groove in an antiparallel orientation (Figure 5; Clouser et al., 2019; Baughman et al., 2020). This interaction is like the NTR distal motif and the NTR β2 strand binding within the groove (Figure 4). The outer residues of the IXI/V motif fit into the holes of the hydrophobic groove due to the kink created by the center proline-182 residue (Freilich et al., 2018; Klevit, 2020). This is described as an extended formation of the CTR in the trans-conformation as the canonical proline can undergo cis-trans isomerization about the I-P peptide bond (Alderson et al., 2017). The interaction is found to occur within the same dimeric subunit and between adjacent dimers as well (Reid Alderson et al., 2021). The lowly populated residual β-strand structure may further encourage binding between the ACD and CTR and other binding partners (Alderson et al., 2017). The IXI/V motif is considered a short linear motif (SLiM) that modulates interactions between proteins (Reid Alderson et al., 2021). In the case of Hsp27, those interactions are between the CTR, the ACD, and possibly client proteins (Reid Alderson et al., 2021). A crystal structure of the truncated Hsp27 ACD co-crystallized with a peptide mimicking the C-terminal IXI/V motif has been solveds (Figure 5; Hochberg et al., 2014). This assembly shows the C-terminal SLiM in an antiparallel alignment to the β8 strand. The binding of the CTR IXI/V motif within the hydrophobic groove of the ACD acts to further stabilize high molecular weight homo-oligomers of Hsp27. It accomplishes this by transiently linking neighboring dimers indicating that the population of bound and unbound IXI/V motifs is mixed (Boelens, 2020). The binding combinations for these regions occur within a dimer and between adjacent dimers. The transient nature of these interacting regions creates a flexible protein that is unexpectedly stabilized. Hsp27 is a dynamic protein that is continuously exchanging its binding regions allowing for substrate capture.

### Hsp27 Regions Involved in Substrate Capture

The three regions of Hsp27 have distinct roles in substrate capture (Jaya et al., 2009; Hochberg et al., 2014; Mainz et al., 2015). The ACD is thought to bind proteins that aggregate *via* the amyloid fibril aggregation pathway (Bakthisaran et al., 2015; Mainz et al., 2015; Freilich et al., 2018; Santhanagopalan et al., 2018; Webster et al., 2019). Client proteins that aggregate *via* this mechanism tend to form β-sheet conformations that resemble β-amyloid fibrils so the attraction between the ACD and the amyloid fibril-like substrate is robust (Mainz et al., 2015; Liu et al., 2020). The neuronal protein α-synuclein is an established substrate of Hsp27 that aggregates *via* the amyloid fibrillar pathway. It was found that full-length Hsp27 and truncated ACD transiently bind monomers of α-synuclein preventing their formation into fibrils (Cox et al., 2016, 2018; Jia et al., 2019). The truncated ACD displayed a lower binding affinity for α-synuclein monomers than the WT indicating that the α-synuclein monomer binding is in large part through the NTR (Jia et al., 2019). Additionally, the isolated ACD of Hsp27 cannot bind to α-synuclein fibrils that are already aggregated (Cox et al., 2018; Selig et al., 2020). Co-sedimentation experiments of Hsp27 lacking the CTR and in the presence of α-synuclein fibrils confirmed that the CTR is required to bind the fibrils (Selig et al., 2020). Therefore, involvement of the NTR and CTR is required when binding fibrillar aggregates of α-synuclein to prevent their elongation (Cox et al., 2018; Selig et al., 2020). These experiments suggest that the ACD alone is not sufficient to protect against amyloidogenic aggregation and that the involvement of all three regions is required.

Further evidence suggests that there is a strict interplay between the three Hsp27 regions to regulate chaperone activity (Baughman et al., 2018, 2020; Freilich et al., 2018). It has been observed that Hsp27 binds Tau, another protein that aggregates *via* the amyloidogenic pathway (Abisambra et al., 2010; Baughman et al., 2018, 2020; Freilich et al., 2018). This is mediated by an interaction between an IXI/V motif of Tau and the ACD hydrophobic groove suggesting that the groove provides protective benefits against fibril formation (Freilich et al., 2018). However, this study also revealed that using truncated forms of Hsp27 lacking the NTR completely abolished this protective activity, further complicating the mechanism by which Hsp27 binds Tau (Freilich et al., 2018). One study suggested that the chaperone activity of Hsp27 extends beyond the ACD hydrophobic groove and is actually dependent on interactions of NTR to client protein (Baughman et al., 2020). It was subsequently determined that a terminal peptide representing the first 13 residues of the Hsp27 NTR binds the ACD hydrophobic groove that binds Tau 50 residues downstream of the highly disordered NTR region (Clouser et al., 2019). These experiments demonstrate a possibility that a binding competition for the ACD groove exists beyond IXI/V-containing proteins such as Hsp27 CTR, Tau, PCBP1, and others. Mutation of the B4/B8 hydrophobic groove to discourage binding between the groove and any IXI/V motif showed an unexpected increase in chaperone activity towards Tau (Freilich et al., 2018). This suggests that the ACD region is not the actual site for chaperoning activity (Baughman et al., 2020). Instead, there may be a competition between the NTR, CTD, and other IXI/V containing entities for the hydrophobic groove of Hsp27. Therefore, the role of the ACD hydrophobic pocket may be to act as a regulatory limiting factor that controls the extent to which Hsp27 binds and chaperones proteins (Chalova et al., 2014; Freilich et al., 2018; Santhanagopalan et al., 2018). In summary, Hsp27 can protect a large array of structurally diverse client proteins by utilizing its dynamic conformational variability. Mutations of Hsp27 can easily disturb this tightly regulated balance and lead to neurodegenerative disorders.

### Missense Mutations Associated With CMT2F and dHMN2B

Mutations within all three regions of Hsp27 are implicated in the neurodegenerative disorders CMT2F and dHMN2B (Arbach et al., 2017; Muranova et al., 2019, 2020; Vendredy et al., 2020). As previously mentioned, there is an abundance of clinical and genetic overlap between the two neuropathies further complicating the elucidation of disease progression. The NTR mutants (Table 1) are associated with dHMN and cause overstabilization of oligomers accompanied by a slight increase in size (Muranova et al., 2015). These mutants have decreased chaperoning activity and are resistant to phosphorylation (Nefedova et al., 2013b; Muranova et al., 2015). The NTR is a dynamic region that is constantly undergoing conformational modifications to accommodate oligomerization and substrate binding. Amino acid changes within this region directly affect the interaction of the NTR with the ACD. The effects are either enhanced or diminished binding to the ACD, both of which have negative consequences that can have a direct impact on the oligomerization, phosphorylation, and chaperoning activity since the three are interdependent. Furthermore, mutations of the NTR could potentially result in diminished chaperone activity toward Tau and α-synuclein. Experimental evidence suggests that the NTR is critical in the effective chaperoning of Tau and α-synuclein (Baughman et al., 2020; Selig et al., 2020). Perhaps disease mutations of the NTR could result in diminished chaperone activity of these proteins aggravating the progression of AD and PD. Several models demonstrate that Hsp27 is critical in preventing the formation of α-synuclein inclusions further supporting the notion that ineffective chaperoning will exacerbate disease progression (Cox et al., 2016, 2018; Cox and Ecroyd, 2017).

**Table 1:** Hsp27 mutations associated with CMT2F and dHMN2B.

Domain/Motif	Mutation	Structural change	Possible consequences	References
NTR after SRLFDQxFG motif	G34R	Large negatively charged residue reduces flexibility	Reduces interaction of NTR (25–37) with dimer interface groove	Muranova et al. (2015) and Clouser et al. (2019)
NTR	P39L	Substitution to L increases flexibility	Either increase in α-helical structure or NTR-NTR interactions	Muranova et al. (2015), Kalmar et al. (2017), and Clouser et al. (2019)
NTR	E41K	Negative charged residue replaced with positive charge residue	Could disrupt contacts made with ACD	Muranova et al. (2015)
NTR β2	G84R	Large negatively charged residue reduces flexibility	Reduces interaction of NTR (74–91) with dimer interface groove	Nefedova et al. (2013b), Nefedova et al. (2017), and Clouser et al. (2019)
ACD β3	L99M	Replacement of L sidechain could disturb contacts with R140	Possibly disturbs the stability of ACD	Nefedova et al. (2013b) and Nefedova et al. (2017)
ACD β5	R127W	Replacement of R abolishes 3 H-bonds with H103 and E108 in L3/4 loop	Could destabilize structure of ACD	Almeida-Souza et al. (2010), Almeida-Souza et al. (2011), Holmgren et al. (2013), and Weeks et al. (2018)
ACD β6 + 7	S135F	Replacement of polar S with hydrophobic F	Could cause solubility issues	Almeida-Souza et al. (2010), Kalmar et al. (2017), Schwartz et al. (2018), and Weeks et al. (2018)
ACD β6 + 7	R136W	Replacement of R abolishes H-bonds with H124 of β5 of the same monomer	Introduction of another large aromatic residue in the crowded floor of the dimer interface groove could cause destabilization	Almeida-Souza et al. (2010), Almeida-Souza et al. (2011), and Weeks et al. (2018)
ACD β6 + 7	R140G	Substitution to G abolishes intradimer contacts. Additionally, an intradimer salt bridge with N129 is disrupted.	Possibly causes destabilization and collapse of the dimer interface groove	Nefedova et al. (2013a), Nefedova et al. (2017), and Kalmar et al. (2017)
ACD β6 + 7	K141Q	Replacement of K results in loss of positive charge and interaction with E126 in β5 of the adjoining monomer.	Could affect the dynamic contacts that are made with the NTR and the CTR	Nefedova et al. (2013a) and Nefedova et al. (2017)
ACD L7/8	T151I	Replacement of T with a hydrophobic residue at the β4/β8 entrance	The introduction of a hydrophobic residue could disturb binding interactions with the groove	Almeida-Souza et al. (2011)
ACD β9	T164A	Replacement of T with a hydrophobic residue	Alteration of the last ACD strand could affect the orientation of the CTR and cause negative effects on interdimer contacts	Chalova et al. (2014)
CTR IXI/V	T180I	Increases hydrophobicity of CTR	Minimal effects	Chalova et al. (2014)
CTR IXI/V	P182L	Substitution with L abolishes rigidity imposed by the center P	Affects binding of IXI/V to groove	Holmgren et al. (2013), Geuens et al. (2017), and Reid Alderson et al. (2021)
CTR IXI/V	P182S	Substitution with S abolishes rigidity imposed by the center P	Affects binding of IXI/V to groove	Chalova et al. (2014) and Nefedova et al. (2017)
C-terminal Extension	R188W	Substitution of charged R with large and hydrophobic W	This may affect the solubility of Hsp27/substrate complexes lead to co-precipitation	Chalova et al. (2014)

The ACD is considered a hotspot for mutations that result in CMT2F and dHMN2B (Kalmar et al., 2017). Mutants R127W, S135F, and R136W destabilize the dimer interface groove resulting in increased monomerization that correlates with increased chaperoning activity (Almeida-Souza et al., 2010; Weeks et al., 2018; Alderson et al., 2019). These mutants were shown to be hyperactive and displayed elevated binding to α and β tubulin (Almeida-Souza et al., 2010, 2011; Weeks et al., 2018). This increased binding was correlated with an increased stability of the microtubule network and is an unfavorable consequence since the dynamic polymerizing nature of microtubules is essential to its function (Almeida-Souza et al., 2011; Kevenaar and Hoogenraad, 2015). Hsp27 mutations R140G and K141Q were found to decrease the thermal stability of the oligomers (Nefedova et al., 2013a). This causes changes in the oligomerization and chaperoning ability of Hsp27 as the two are connected. The T151I mutant occurs at the end of the β4/β8 groove within an α-helix and surprisingly did not affect chaperone activity and did not have increased binding to tubulin/microtubules (Almeida-Souza et al., 2010; Klevit, 2020). The T164A mutant was shown to cause dissociation of oligomers accompanied by a decrease in thermal stability (Chalova et al., 2014). The ACD mutations lead to destabilization of the secondary structure at the dimer interface. This may lead to aberrations within the dimer interface groove that disrupt contacts made by the NTR. If the NTR is not regulated properly by the ACD, changes to the quaternary structure and chaperoning ability arise. It was demonstrated that mutations of the ACD to enhance the release of the NTR increased Tau substrate capture (Baughman et al., 2020). Disease mutations of the ACD could strengthen the interaction between the NTR and the ACD. The point mutations of the ACD occur at the dimer interface groove and one near the β4/β8 groove, both regions known to transiently bind stretches of the NTR. A mutation from a charged residue to a hydrophobic residue could amplify the interaction between these ACD binding regions and the hydrophobic NTR resulting in a protein that can no longer effectively chaperone Tau.

Lastly, mutations in the CTR and extension are causative for dHMN. The T180I mutant significantly improves the stability of Hsp27 oligomers and does not alter chaperoning activity (Chalova et al., 2014). However, this mutation could impact the interconversion rate between cis and trans states about the I180-P182 bond (Alderson et al., 2017). Disturbances near this region could explain the increased oligomer stability that is not conducive to the mobile nature of Hsp27. The proline at position 182 is the central residue of the IXI/V motif and is susceptible to two different mutations, P182L and P182S. These mutations, as listed in Table 1, result in an increase in oligomerization resulting in aggregation and diminished chaperone activity (Chalova et al., 2014). The P182L mutation similarly results in very large oligomers that have diminished chaperone activity. This was confirmed both *in vitro* and *in vivo* with dHMN patient-derived stem cells (Reid Alderson et al., 2021). It was found that the P182L mutant decreases the affinity of the IXI/V for the β4/β8 groove possibly due to the disruption of the trans configuration of the proline residue which is evidenced to facilitate binding to the ACD (Alderson et al., 2017; Reid Alderson et al., 2021). This liberates the interaction between the CTR and ACD and results in increased binding of the NTR to the ACD groove (Reid Alderson et al., 2021). This explains why the P182L and possibly the P182S result in large oligomers that are unable to chaperone client proteins. The NTR regions are erroneously bound to the ACD limiting their availability to chaperone, resulting in large oligomeric complexes (Freilich et al., 2018; Reid Alderson et al., 2021). This study further revealed that other IXI/V-containing proteins have the potential to bind the Hsp27 hydrophobic groove. One such protein is poly(rC)-binding protein 1 (PCBP1; Geuens et al., 2017; Reid Alderson et al., 2021). The single-stranded nucleic acid binding protein has several neuronal transcript targets suggesting that disruption of PCBP1 may have deleterious effects on downstream proteins that are vital for neuronal activity (Geuens et al., 2017). P182L bound with higher affinity to PCBP1 compared to the WT when using a mutant cell line (Geuens et al., 2017). The increased interaction between the Hsp27, P182L, and PCBP1 caused an overall reduction in translational repression of PCBP1, resulting in elevated protein expression levels (Geuens et al., 2017). The R188W mutation does not alter oligomeric size or stability but does have a significant effect on chaperoning activity (Chalova et al., 2014). The substitution of a charged amino acid with a large hydrophobic residue may affect the solubility of Hsp27/substrate complexes (Chalova et al., 2014). In addition, it is suspected that the R188 mutation could interfere with the formation of the residual β-strand structure formed by residues 193–199 which is hypothesized to regulate oligomer and substrate dynamics (Alderson et al., 2017).

### Concluding Remarks

Hsp27 plays a central role in several physiological processes, including maintaining cytoskeletal dynamics, apoptosis, signal transduction, activation of the proteasome, and protein folding (Parcellier et al., 2005; Garrido et al., 2012). It is comprised of several structurally distinct regions, including the NTR, the ACD, and the CTR. The NTR region is primarily responsible for substrate capture with sequences that are equipped to bind substrates. The NTR is generally bound to the ACD, which regulates the NTR, so it is not continuously and arbitrarily fishing for the substrate. The CTR regulates this interaction between the NTR and the ACD by competing with binding sites on the ACD that then render the NTR available for substrate capture. This delicate interplay is disturbed by a myriad of point mutations that cause neurodegenerative diseases. These mutations affect the three key regions of Hsp27 in both divergent and overlapping fashions, thus further complicating our current understanding of disease progression of CMT2F and dHMN2B. Their role in pathological progression may lie in the consequential inability to protect vital neuronal proteins. Similarly, the inability to properly chaperone these client proteins may worsen diseases such as Alzheimer’s and Parkinson’s disease. Although there have been many breakthroughs during the investigation of these relationships, further work is required to better elucidate the link between disrupted Hsp27 structure and the progression of these debilitating neuropathies. Furthermore, subsequent efforts are required to better understand how the conformational variability of Hsp27 regulates oligomerization, phosphorylation, and substrate capture with respect to Hsp27’s encompassing role in human physiology.

## Author Contributions

BH wrote and prepared the manuscript. RB, ZH, and BH contributed to the editing of the manuscript at all points of the preparation process. All authors contributed to the article and approved the submitted version.

## Conflict of Interest

The authors declare that the research was conducted in the absence of any commercial or financial relationships that could be construed as a potential conflict of interest.

## Publisher’s Note

All claims expressed in this article are solely those of the authors and do not necessarily represent those of their affiliated organizations, or those of the publisher, the editors and the reviewers. Any product that may be evaluated in this article, or claim that may be made by its manufacturer, is not guaranteed or endorsed by the publisher.
